# Impact of postoperative cerebral complications in acute infective endocarditis: a retrospective single-center study

**DOI:** 10.1186/s13019-024-02768-x

**Published:** 2024-04-20

**Authors:** Kayo Sugiyama, Hirotaka Watanuki, Masato Tochii, Takayuki Kai, Daisuke Koiwa, Katsuhiko Matsuyama

**Affiliations:** Department of Cardiac Surgery, Aichi Medical University Hospital, 1-1 Yazako Karimata, Nagakute, 480-1195 Aichi Japan

**Keywords:** Infective endocarditis, Cerebral infarction, Cerebral hemorrhage, Mycotic aneurysm, Subarachnoid hemorrhage

## Abstract

**Background:**

The treatment of patients with infective endocarditis (IE) who have preoperative cerebral complications remains less understood. Therefore, this study aimed to retrospectively evaluate the clinical outcomes of patients with acute IE based on preoperative intracranial findings.

**Methods:**

Of 32 patients with acute IE treated at our hospital between August 2015 and March 2022, 31 patients of whom preoperative intracranial imaging evaluation was available were included in our analysis and compared with those with and without intracranial findings. We controlled the mean arterial blood pressure and activated clotting time (ACT) to prevent abnormally high perfusion pressures and ACTs during cardiopulmonary bypass (CPB). The preoperative background, and postoperative courses focusing on postoperative brain complications were reviewed.

**Results:**

Among the 31 patients, 20 (65%) had preoperative imaging findings. The group with intracranial findings was significantly older, with more embolisms in other organs, positive intraoperative pathology findings, and longer CPB times. A new cerebral hemorrhage developed postoperatively in one patient without intracranial findings. There were no early deaths; two patients had recurrent infections in each group, and one died because of sepsis in the late phase in the group with intracranial findings.

**Conclusions:**

Positive intracranial findings indicated significantly active infectious conditions preoperatively but did not affect the postoperative course. Patients without preoperative cerebral complications can develop serious cerebral hemorrhage. Although meticulous examination of preoperative cerebral complications in all patients with IE is essential, a strategy should be adopted to prevent cerebral hemorrhage, even in patients without intracranial findings.

## Background

Cerebral complications occur in 20–40% of patients during the active course of infective endocarditis (IE) and can be associated with poor clinical outcomes [1, 2]. Particularly, cerebral hemorrhage may worsen the degree of brain injury due to the use of anticoagulants during cardiopulmonary bypass (CPB), which can cause devastating brain damage with poor prognosis [3]. The appropriate timing of surgery for patients with IE who have preoperative cerebral complications remains controversial [4]. Guidelines from the Society of Thoracic Surgeons recommend delaying surgery for at least 4 weeks when patients have experienced major ischemic strokes or intracranial hemorrhage [5, 6]. In addition, several recent studies have reported that the risk of neurological impairment is lower than that estimated in patients with IE who have cerebral infarctions and have undergone early surgery [7, 8]. However, it may be more difficult to determine the optimal timing of surgery in patients with intracranial hemorrhage [9–11].

Recently, advances in neuroimaging technology have led to the detection of silent intracranial findings, and their incidence is significantly higher than expected, at approximately 80% [12–15]. The benefits of diagnostic imaging have enabled more detailed preoperative evaluation of patients and the determination of the timing of surgery. However, it is also important to note that mycotic aneurysms are frequently located in the peripheral arteries, as they are too small to be detected using computed tomographic angiography (CTA) or magnetic resonance angiography (MRA) [13, 16, 17]. In accordance with Hess et al.’s findings, the radiological description of cerebral lesions highlighted cortical cerebral microbleeds and small watershed ischemic lesions as the most frequent pattern in the population studied [13]. Moreover, cerebral hemorrhage can occur even without obvious preoperative cerebral lesions. Intracranial hemorrhage of undetermined etiology can also result from septic arteritis, with erosion of the vessel wall caused by ‘‘microemboli’’ but without a well-delineated aneurysm [18, 19].

Furthermore, the relationship between intracranial hemorrhage and anticoagulation therapy is not well established. Ota et al. [20] successfully managed CPB by continuous administration of nafamostat mesylate. To decrease the tendency toward hemorrhage, some researchers have suggested that reduced heparinization combined with a heparin-coated pump system would be useful during cardiac surgery [16]. However, the relative importance of blood pressure and pump flow in patients with intracranial hemorrhage as determinants of cerebral perfusion during CPB remains unclear.

Here, we retrospectively evaluated the clinical outcomes of patients with acute IE by categorizing them into those with and without intracranial findings based on preoperative imaging evaluation. Notably, at our institute, heparin dosage is adjusted to avoid over extension of activated clotting time (ACT), and ACT is strictly controlled with a target of approximately 500 s. In addition, steps are taken to prevent abnormally high perfusion pressures during CPB.

## Methods

### Study design

**Fig. 1:**
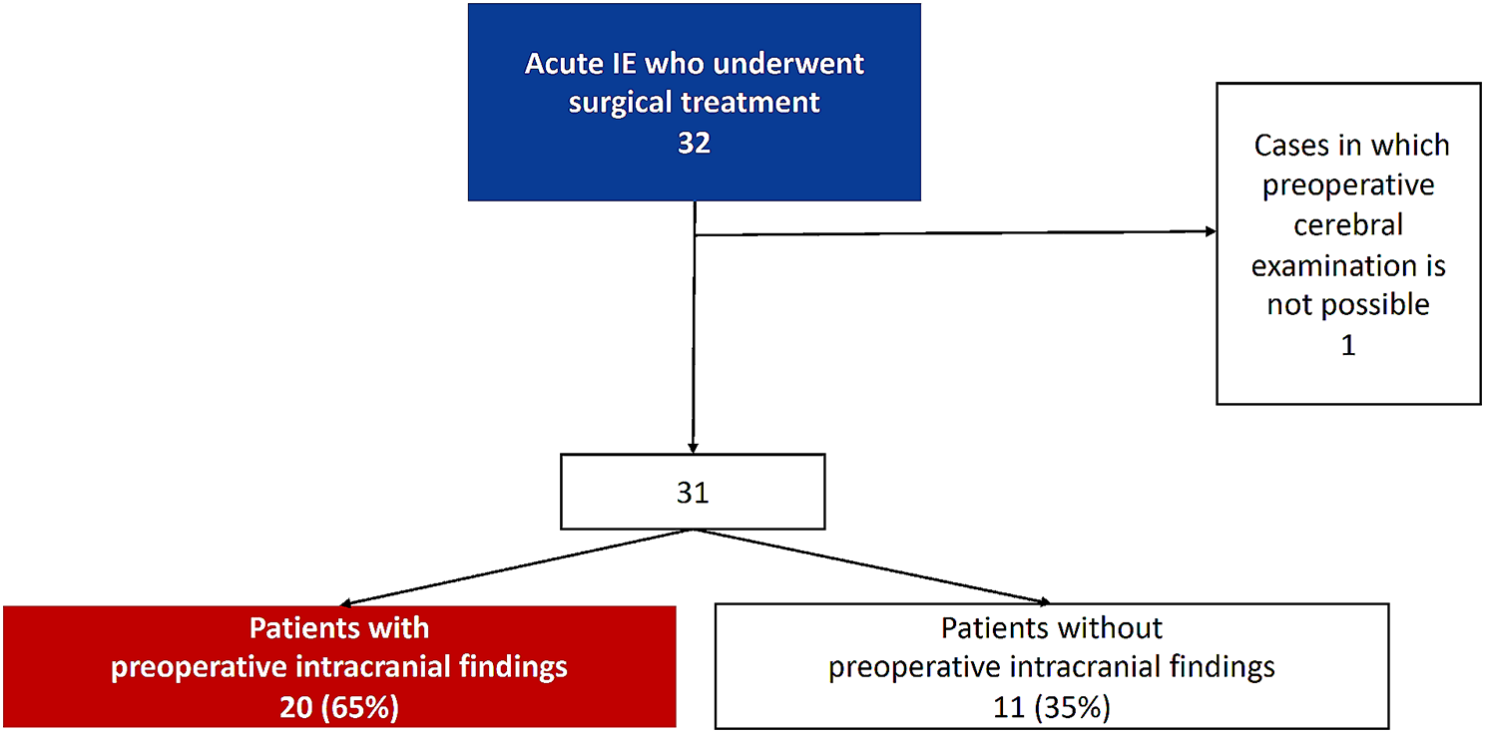
Patient selection

We retrospectively reviewed 32 cases of acute IE treated at our hospital from August 2015 to March 2022 (Fig. 1). In the 31 cases in which preoperative cerebral examinations were performed, we examined preoperative patient background, intraoperative CPB management, and postoperative course based on with or without preoperative intracranial findings. Preoperative intracranial findings were defined as those with or without positive findings from brain magnetic resonance imaging (MRI), MRA, or computed tomography angiography (CTA). All patients had been diagnosed with acute IE according to modified Duke criteria [21, 22]. The exclusion criteria included IE that healed in the chronic phase and right-sided IE that did not involve the left-side valves. In addition, one patient who did not undergo preoperative brain MRI because of cardiogenic shock was excluded. Thirty-one patients were categorized into those with (*n* = 20) and without (*n* = 11) preoperative intracranial findings.

The following patient demographics and comorbidities were recorded: age, sex, diabetes mellitus, chronic renal disease requiring hemodialysis, immunosuppressive drug administration, history of cardiac surgery, history of coronary artery disease, history of cerebrovascular disease, and whether it was an emergency case. The following preoperative data related to IE and cardiac function were also collected: duration from onset to diagnosis, duration from diagnosis to surgery, causative microorganisms, thrombi in other organs, and preoperative echocardiographic data. Other organ embolisms were excluded from CT. Based on blood culture results, antibiotics were administered according to the recommendations of the infection control team at our institute. Our patients received intravenous antibiotics for at least 4 weeks preoperatively and 4–6 weeks postoperatively. Postoperative antibiotics were administered for either 6 weeks if the intraoperative culture was positive or 4 weeks if it was negative. Based on intraoperative pathological findings, a case was classified as pathologically “active” if either of the following was observed in the excised valve: (1) an acute inflammatory reaction microscopically or (2) organisms found microscopically [23]. Major adverse cardiac and cardiovascular events (MACCE) were the primary endpoints. MACCE were defined as the composite of all-cause mortality, hospitalization due to heart failure, repeat cardiac surgery, and brain-related events, including cerebral hemorrhage or stroke. Secondary endpoints were IE-related postoperative events related to IE, recurrence, and adverse events related to recurrent IE.

An intracranial mycotic aneurysm was diagnosed based on the CTA or MRA results. Preoperative intracranial findings were defined as fresh lesions from a cerebral infarction, cerebral hemorrhage, subarachnoid hemorrhage, or intracranial mycotic aneurysms. In addition, pre-and postoperative symptoms related to neurological deficiency included deep coma, disturbances in consciousness, hemiplegia, and speech impediments. Deficits that persisted until hospital discharge were designated as permanent neurological deficits. Delayed awakening, transient loss of orientation, slurred language, poor response to commands, and transient hemiparesis that resolved by hospital discharge were designated as temporary neurological deficits. In the case of intracranial mycotic aneurysms, catheter-based cerebral angiography was performed if required. Although intracranial aneurysms with a maximum diameter > 5 mm were considered for endovascular treatment, the decision to clip or resect the aneurysms depended on the neurosurgeon. Therefore, in patients with ruptured mycotic aneurysms, neurosurgery or endovascular surgery should be initially performed when necessary, and cardiac surgery should be postponed for at least 4 weeks with adequate antibiotic therapy [5, 6].

### CPB and anticoagulation procedure

Indications for valve surgery included heart failure unresponsive to medical therapy, persistent infection, repeat embolization, high embolic risk, and perivalvular extension of the IE. All surgeries were performed using routine procedures except for CPB anticoagulation and perfusion flow management. Anticoagulation during CPB was managed as follows: before connecting to the extracorporeal circuit, 100 IU/kg of heparin (our normal dosage for CPB:300 IU/kg) was administered to obtain an ACT ˃ 300 s. The ACT was targeted at approximately 500 s during CPB (our standard range was 400 s or longer, with no upper limit). The target mean arterial blood pressure during cardiopulmonary bypass was approximately 50 mmHg. The maximum intraoperative ACT and mean arterial blood pressure during CPB were measured. Continuous bilateral cerebral regional oxygen saturation values were monitored using the INVOS™ Cerebral/Somatic Oximetry Adult Sensors (Medtronic, Minneapolis, MN, USA) were also recorded and assessed.

### Statistical procedures

Continuous and categorical variables are expressed as mean ± standard deviation or median (range) and number (%) of patients, respectively. Categorical variables were analyzed using Fisher’s exact test, continuous variables were compared using Student’s t-test, and non-parametric variables were analyzed using the Mann–Whitney U test. All data analyses were performed using the JMP 17.1 software (SAS Institute, Cary, NC, USA). Statistical significance was set at *p* < 0.05.

## Results

Table 1 summarizes the preoperative clinical characteristics of the 31 patients. Of the 31 patients, 16 (52%) were male. The median age of the patients was 67 years (range, 32–82 years).

**Table 1:** Characteristics of patients

		with cerebral complications *n* = 20	without cerebral complications *n* = 11	p value
Age		71 (42–82)	54 (32–73)	0.0053
Sex (male, %)		10 (50)	6 (55)	0.81
Previous cardiac surgery (%)		4 (20)	0	0.051
Prosthetic valve endocarditis (%)		4 (20)	0	0.051
Diabetes mellitus (%)		2 (10)	2 (18)	0.52
Use of immunosuppressive drugs (%)		3 (15)	1 (9)	0.63
Hemodialysis (%)		1 (5)	0	0.34
Preoperative ejection fraction (%)		65 (25–80)	65 (55–75)	0.88
Embolism to other organs (%)		11 (55)	2 (18)	0.040
Blood culture (%)		19 (95)	11 (100)	0.34
	Staphylococci (%)	4 (20)	3 (27)	0.56
	Streptococcus (%)	15 (75)	8 (73)	
Duration from onset of symptoms to diagnosis (days)		16 (1-213)	19 (1-150)	0.70
Duration from diagnosis to surgery (days)		22 (0-157)	28 (1-143)	0.24

No significant differences were observed between the two groups in sex, duration from symptoms to diagnosis or from diagnosis to surgery, causative organisms, diabetes mellitus, hemodialysis, use of immunosuppressive drugs, previous cardiac surgery, history of ischemic heart disease, history of cerebral infarction, emergency cases, or preoperative cardiac function. However, the group with preoperative intracranial findings was significantly older (*p* = 0.0053) and had embolisms in other organs (*p* = 0.040). Furthermore, heart failure was significantly more common in the group without intracranial findings, whereas those with intracranial findings had various surgical indications (*p* = 0.025). Including duplicates, of the 20 (65%) patients with preoperative intracranial findings, 19 had fresh infarcts, 6 had cerebral hemorrhages, including 3 subarachnoid hemorrhages, and 6 had intracranial mycotic aneurysms. Four (20%) of the patients in this group had neurological symptoms: two experienced disturbances in consciousness, one had paralysis of the extremities, and one had a speech impediment. Patients in this group were referred for neurosurgery preoperatively; however, none of them required preoperative neurosurgical intervention.

**Fig. 2:**
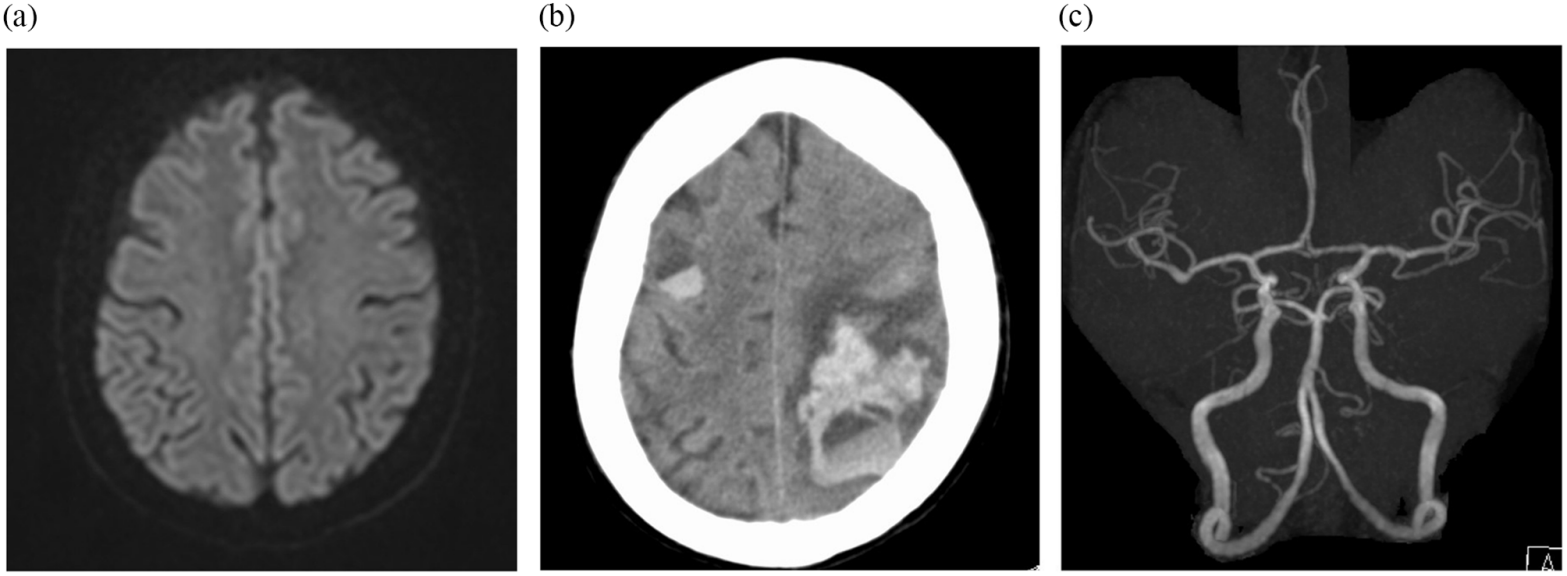
(**a**) Preoperative brain magnetic resonance imaging of a patient showing almost normal. (**b**) Postoperative brain computed tomography image of a patient showing broad cerebral hemorrhage. (**c**) Preoperative brain magnetic resonance angiography of a patient showing no mycotic aneurysm

Table 2 summarizes the operative procedures, intraoperative CPB data, and postoperative outcomes. Nineteen (61%) patients underwent minimally invasive cardiac surgery, and two (6%) required complicated surgical manipulation, including annular repair, due to extensive infection spread to the valve annulus. All patients were uneventfully weaned from CPB and no assistance device was required. Intraoperative pathology specimens revealed active IE in 24 patients (77%). No early postoperative deaths were observed; however, one patient (3%) in the group without preoperative intracranial findings (Fig. 2a, 2b) developed extensive cerebral bleeding (Fig. 2c), requiring tracheostomy. The patient recovered to almost normal neurological status after meticulous rehabilitation. The median follow-up period was 421 days (range, 20–2030 days). During the follow-up, MACCE occurred in four (13%) cases, of which one patient with preoperative intracranial findings died in the late period because of sepsis, one without preoperative intracranial findings developed a transient ischemic attack, and two in each group needed redo open-heart surgery. These patients underwent mitral valve replacement for the recurrence of severe mitral valve regurgitation but not for IE recurrence. Considering IE-related postoperative events in five (16%) cases, during follow-up, sepsis caused by recurrent IE developed in three patients: one with preoperative intracranial findings and two without preoperative intracranial findings. One patient with preoperative intracranial findings developed a ruptured mycotic splenic artery aneurysm, which was treated with interventional radiology.

**Table 2:** Operative procedures and outcomes

		with cerebral complications *n* = 20	without cerebral complications *n* = 11	p value
Operative indication	Heart failure unresponsive to medical therapy (%)	8 (40)	10 (91)	0.025
	High embolic risk (%)	8 (40)	1 (9)	
	Persistent infection (%)	2 (10)	0	
	Perivalvular extension (%)	2 (10)	0	
Emergency surgery (%)		7 (35)	1 (9)	0.086
Minimally invasive cardiac surgery (%)		11 (55)	8 (73)	0.33
Operation time (minutes)		372 (210–792)	247 (219–452)	0.085
CPB (minutes)		198 (101–410)	177 (82–248)	0.046
ACC (minutes)		115 (58–213)	94 (46–136)	0.071
Maximum ACT (seconds)		502 (388–628)	485 (405–569)	0.96
Maximum mean arterial blood pressure (mmHg)		63 (49–78)	70 (50–87)	0.0011
ICU stay (days)		3 (1–28)	2 (2–15)	0.84
Hospitalization (days)		29 (14–43)	15 (8–84)	0.23
Neurological outcomes (%)		1 (5)	1 (9)	0.66
Tracheostomy (%)		1 (5)	1 (9)	0.66
Pacemaker implantation (%)		2 (10)	0	0.18
Renal complication (%)		0	0	
Pathological results (%)		19 (95)	5 (45)	0.0026
Early mortality (%)		0	0	
Late mortality (%)		1 (5)	0	0.34
MACCE (%)		2 (10)	2 (18)	0.52
Events related to infection (%)		2 (10)	3 (27)	0.22

ACC, aortic cross-clamp; ACT, activated clotting time; CPB, cardiopulmonary bypass time; ICU, intensive care unit; MACCE, major adverse cardiac or cerebrovascular events

**Fig. 3:**
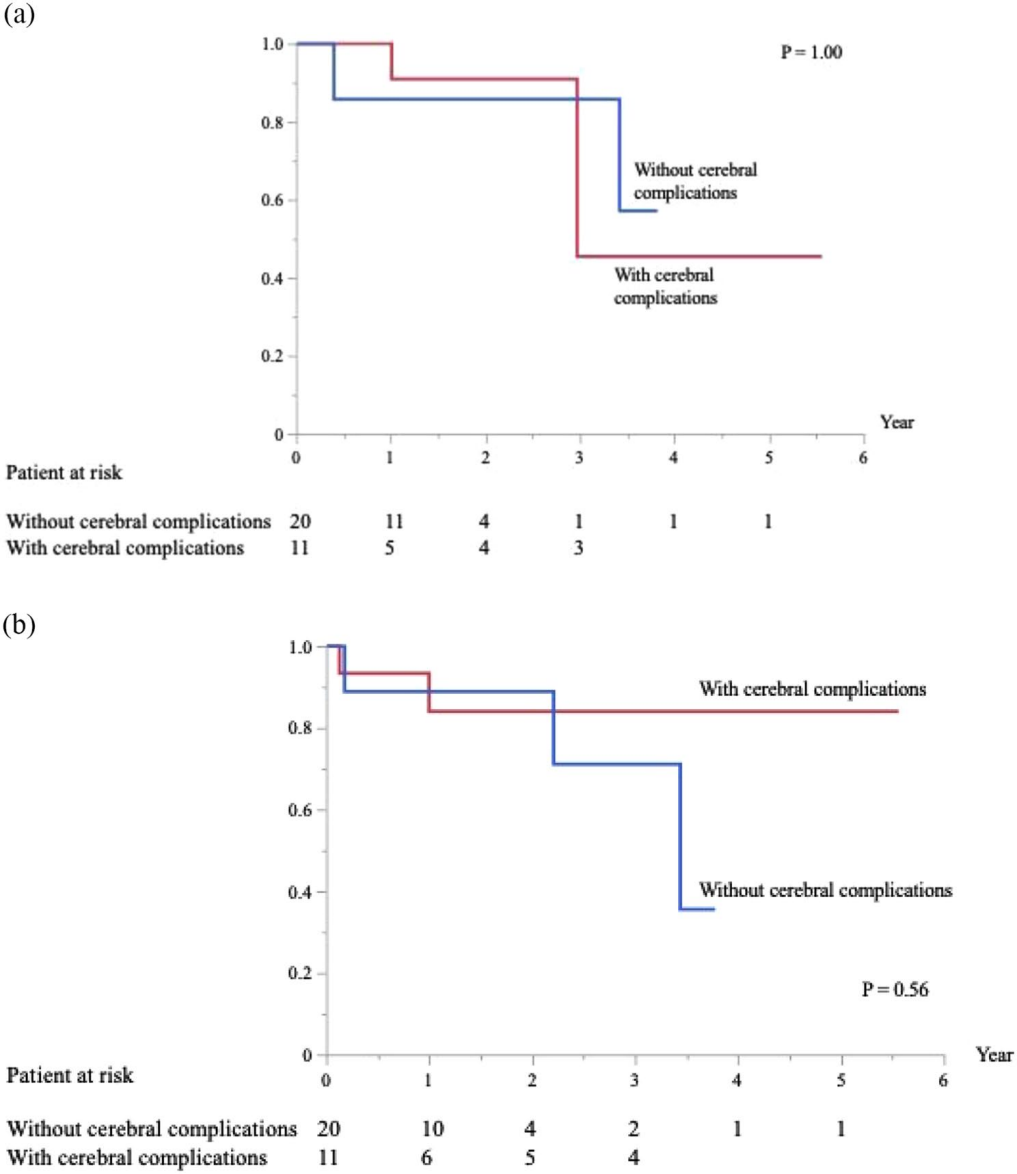
(**a**) Freedom from MACCE in patients with and without cerebral complications. (**b**) Freedom from events related to IE in patients with and without cerebral complications

No significant differences were observed in the minimally invasive cardiac surgery cases, operation time, aortic cross-clamp time, maximum ACT, length of intensive care unit stay, length of hospital stay, early postoperative death, postoperative neurological complications, postoperative cardiac-related complications, late postoperative death, recurrent infection, and MACCE. However, significant differences were observed in CPB time (*p* = 0.046), maximal mean arterial blood pressure (*p* = 0.0011), and pathological results (*p* = 0.0026) between the two groups. The Kaplan–Meier curves showed no significant difference in MACCE between the two groups (*p* = 1.0; Fig. 3a). The Kaplan–Meier curves showed no significant difference in events related to IE between the two groups (*p* = 0.56; Fig. 3b).

## Discussion

In this study, no early postoperative death or new cerebrovascular complications occurred in the group with preoperative intracranial findings. In contrast, a new cerebral hemorrhage developed in one patient whose preoperative MRI showed neither infarction nor hemorrhage. These results indicate that preoperative imaging alone does not predict postoperative cerebral complications and that positive preoperative findings are not immediately associated with postoperative cerebral complications. Although the number of cases is small, our CPB strategy has allowed the avoidance of serious cerebral hemorrhage even in cases with preoperative cerebral complications.

Recently, silent intracranial findings have been detected because of advances in neuroimaging technology, and their incidence is much higher than expected, at approximately 80% [12–15]. Despite the improved accuracy of head MRI, detection of microscopic emboli, vascular injuries, and disruptions remains difficult. Furthermore, blood vessels are known to become fragile in IE [13, 16–19], and cerebral hemorrhage can occur even in the absence of an apparent aneurysm, requiring more attention than in ordinary cardiac surgery. Intracranial hemorrhage caused by small-vessel vasculitis might have a higher mortality than ruptured intracranial infectious aneurysm and hemorrhage after ischemic stroke [24]. Thus, the optimal timing of cardiac surgery may differ depending on the mechanism of the hemorrhage. Some reports proposed the possibility of not delaying cardiac surgery in patients with asymptomatic neurological complications because there is no increase in the postoperative exacerbation rate or mortality [13, 14, 24]. Thuny et al. reported that in patients with IE, mortality and neurological outcome depend on the type of cerebral complication [25]. They reported that although patients with stroke have a significant excess mortality, particularly in case of mechanical prosthetic valve IE or impaired consciousness, those with silent cerebral complications or transient ischemic attack have a relatively good prognosis [25]. Moreover, they described that even if valvular surgery can exacerbate cerebral damage after cerebral complications, the risk of postoperative neurologic exacerbation appears to be low after silent cerebral complications, transient ischemic attack, and non-massive ischemic stroke [25]. Based on the results of our study, while the number of cases was small, we believe that only considering cases with preoperative neurological symptoms and intracranial findings, as described in the above reports, is not appropriate. Cooper et al. reported that the mortality rate at 3 months was similar among patients with clinical stroke and patients with asymptomatic stroke, whereas the rate was significantly lower among patients with no MRI evidence of acute brain embolization [12]. This suggests that the implications of acute brain embolization for the treatment of IE, particularly with regard to surgical intervention, may be independent of the clinical manifestation of the embolic event [12]. Therefore, although it would be beneficial if more detailed tests were developed, suspecting preoperative cerebral complications in all IE cases is crucial. It is worth mentioning that Venn et al. proposed an algorithm for preoperative management of patients with IE and hemorrhagic neurological complications, which depends on the mechanism and severity of the hemorrhage regardless of symptoms [26].

In this study, the group with intracranial findings was significantly older, had more positive intraoperative pathological findings, and had more organ embolisms. Cerebral complications occur during the active course of IE [1, 2]. The risk of embolism is reportedly greatest within 14 days of initial IE diagnosis [5]. Furthermore, patients with intracranial hemorrhage reportedly have higher rates of other symptomatic systemic embolisms and mycotic aneurysms [24]. Therefore, prompt initiation of antibiotic therapy is the most effective strategy for reducing the rate of septic embolism [2]. Nevertheless, making a rapid diagnosis or initiate antimicrobial therapy is often not possible. In this study, no significant differences were observed in the timing of IE diagnosis or surgery and further, as expected, the group with intracranial findings showed more active infection before surgery compared to those without. However, no significant differences were observed in most CPB data and postoperative courses. Appropriate management during CPB can result in a desirable postoperative course even in patients with preoperative cerebral complications.

To decrease the tendency toward hemorrhage, reduced heparinization combined with a heparin-coated pump system would be useful during cardiac surgery [16, 20]. Okada et al. reported successful mitral repair with the addition of low-dose heparin and nafamostat mesylate in a young female patient with a recent extensive cerebral infarction due to septic embolization [27]. Ota et al. successfully managed CPB with the continuous use of nafamostat mesylate in patients with acute IE with recent intracranial hemorrhage [20]. However, nafamostat mesylate is difficult to adjust, and its use requires informed consent as well as approval by an ethics committee. In addition, the ACT value fluctuates relative to the increase or decrease in the sustained amount of nafamostat mesylate during CPB; consequently, it cannot be used as an absolute measure, which raises safety concerns. Although achieving statistical significance with a small number of cases is difficult, we are satisfied with the fact that with our strategy, all patients were successfully discharged from the hospital without any postoperative cerebral complications, aside from one patient without preoperative cerebral complications who had serious cerebral hemorrhage. Therefore, our strategy of strict ACT control after heparin administration may be acceptable and feasible for preventing intracranial hemorrhage. In general, the use of anticoagulant therapy and appropriate flow pressure in patients with intracranial hemorrhage remains controversial. Further studies are required to address these issues. Although their reports were based on cases with preoperative cerebral complications, we believe that measures should be taken based on the assumption that postoperative cerebral complications can occur in any case, even with the absence of imaging findings, as mentioned earlier.

Study limitations.

This study had some limitations. First, relatively few patients were included because of the rarity of the condition. Second, this was a retrospective single-center experience lacking any form of randomization. The study had a small number of cases (32) and was not strictly a cohort study. The number of patients with infective endocarditis is limited, and we believe that a multicenter study is needed to address this limitation. However, the variation in preoperative evaluation and use of CPB between centers could account for the difficulty of performing multicenter studies. To solve these problems, we must utilize the same strategy for treatment of patients with IE. Third, the clinical background differs at the time of detection of intracranial findings. For example, intracranial findings are more likely to occur early when the infection is not well-controlled; therefore, findings of infection are more likely to be severe. Similarly, emboli in other organs may be more common, inflammatory findings may be higher, and sepsis and infection findings at the time of surgery may be positive. If more cases are registered, propensity score matching should be performed. Fourth, cases in which MRI could not be performed were excluded from this study, and true severe cases were excluded. Further, current literature does not provide any recommendations on mean arterial blood pressure in patients with intracranial hemorrhage during CPB. Therefore, further studies on the effects of perfusion pressure on cerebral hemorrhage are warranted.

## Conclusion

Patients with positive intracranial findings were in a significantly active infectious condition preoperatively; however, this did not affect their postoperative course. As serious postoperative cerebral complications can develop even in the absence of preoperative intracranial imaging findings, it is extremely important to take precautions against postoperative cerebral complications in all IE cases. We expect that our strategy of maintaining a low intraoperative ACT may be an effective measure; however, further studies related to appropriate mean systemic flow pressure are warranted.

## Data Availability

The datasets used and/or analyzed in the current study are available from the corresponding author upon reasonable request.
